# Kinetics of Periodate-Mediated Oxidation of Cellulose

**DOI:** 10.3390/polym16030381

**Published:** 2024-01-30

**Authors:** Nazmun Sultana, Ulrica Edlund, Chandan Guria, Gunnar Westman

**Affiliations:** 1 Fibre and Polymer Technology, School of Engineering Sciences in Chemistry, Biotechnology, and Health, KTH Royal Institute of Technology, SE-100 44 Stockholm, Sweden; 2 Organic Chemistry, Chemistry, and Chemical Engineering, Chalmers University of Technology, Kemigården 4, SE-412 96 Gothenburg, Sweden; 3 FibRe—Centre for Lignocellulose-Based Thermoplastics, Department of Fibre and Polymer Technology, KTH Royal Institute of Technology, SE-100 44 Stockholm, Sweden; 4 Department of Petroleum Engineering, Indian Institute of Technology (IIT-Indian School of Mines), Dhanbad 826 004, India; 5 FibRe—Centre for Lignocellulose-Based Thermoplastics, Department of Chemistry and Chemical Engineering, Chalmers University of Technology, SE-412 96 Gothenburg, Sweden

**Keywords:** cellulose derivatives, oxidation, cellulose, periodate, dialdehyde, kinetic model

## Abstract

The oxidation of cellulose to dialdehyde cellulose (DAC) is a process that has received increased interest during recent years. Herein, kinetic modeling of the reaction with sodium periodate as an oxidizing agent was performed to quantify rate-limiting steps and overall kinetics of the cellulose oxidation reaction. Considering a pseudo-first-order reaction, a general rate expression was derived to elucidate the impact of pH, periodate concentration, and temperature on the oxidation of cellulose and concurrent formation of cellulose degradation products. Experimental concentration profiles were utilized to determine the rate constants for the formation of DAC (k_1_), degradation constant of cellulose (k_2_), and degradation of DAC (k_3_), confirming that the oxidation follows a pseudo-first-order reaction. Notably, the increase in temperature has a more pronounced effect on k_1_ compared to the influence of IO_4_^−^ concentration. In contrast, k_2_ and k_3_ display minimal changes in response to IO_4_^−^ concentration but increase significantly with increasing temperature. The kinetic model developed may help with understanding the rate-limiting steps and overall kinetics of the cellulose oxidation reaction, providing valuable information for optimizing the process toward a faster reaction with higher yield of the target product.

## 1. Introduction

Cellulose is seen as a significant material feedstock alternative to petroleum-based polymers. Cellulose derivatives have been synthesized for over a century to modify both structure and performance, enabling cellulose valorization in various applications [1]. A renewed and rapidly growing industrial interest in cellulose-modification chemistry and processes has emerged more recently and is important for advancing sustainable production and utilization of cellulose-based materials and chemicals. 

Dialdehyde cellulose (DAC) is a cellulose derivative of growing importance. DAC is obtained by selective oxidation of cellulose repeating units by periodate ions, which cause cleavage of the C2−C3 bonds on the glucose rings of the cellulose backbone, and subsequent formation of aldehyde groups at these positions [2,3,4,5,6]. Generated aldehyde groups can be further oxidized to carboxyl groups [4], reduced to alcohol groups [7], converted to imines (Schiff bases) with primary amines [8,9], or reacted with other agents to achieve crosslinking or functionalization; DAC is, therefore, a potent and viable platform for the preparation of cellulose-based materials with tunable properties [10]. DAC has been explored as a stand-alone material and used as a precursor for the immobilization of functional groups, such as amino or carboxyl groups, enzymes, and antibodies [11]. Functionalized DAC has potential as an adsorbent material in water purification [12]. Pharmaceutical and energy applications of DAC are gaining increasing attention and were recently reviewed [13]. 

Several factors influence the outcome of DAC synthesis, including the degree of crystallinity of cellulose [2], pH of the solution [14], periodate concentration, and temperature [15]. The efficiency of cellulose oxidation and the periodate ions’ reactivity is influenced by increasing the reaction temperature or adding metal salts as activators [16]. An early kinetic study of mild chemical reactions (room temperature, diluted aqueous solution) occurring within cellulose was given by Goldfinger et al. [17] based on accessible regions in the samples, which seem to correspond to the amorphous and crystalline areas. The influence of cellulose crystallinity on periodate oxidation has been well investigated [2,10,16,18]. An increasing degree of oxidation is reported to coincide with a reduction in crystallinity, attributed to the disruption of the ordered packing of cellulose molecules following the opening of the glucopyranose ring [10]. A high degree of crystallinity in the cellulose decreases cellulose oxidation [2,18]. Another challenge faced in cellulose oxidation is the slow kinetics and dilute reaction conditions required for the oxidation, which requires considerable amounts of water and energy [19]. Given the low reactivity of cellulose, achieving high aldehyde contents necessitates the use of a substantial amount of periodate and extended oxidation times, which are not beneficial from a green chemistry perspective [2]. The high temperature and acid can furthermore cause cellulose or dialdehyde cellulose degradation [20]. The choice of reaction conditions involves a potential trade-off between achieving a high product yield and maintaining good reaction efficiency. Furthermore, the formed aldehyde is in equilibrium with the hydrate form, which makes it difficult to accurately quantify the amount of aldehyde formed. Additionally, the aldehyde may cross-link with hydroxyl groups on the cellulose backbone forming hemiacetals. 

Clearly, individual reaction parameters such as temperature or pH impact the final outcome of DAC; however, synergistic effects and intricate interplay between various factors add a layer of complexity to process design. The interaction between temperature, time, and other factors cannot be revealed by the one-parameter-at-a-time approach, where the tuning of one parameter may lead to false optima and neglect interaction effects. A design of experiments (DOE) that allows for a systematic exploration of multiple factors is a powerful tool to reveal such cooperative variations. Kinetic modeling makes use of such multivariate analysis to reveal the factors governing the rate with which a reaction proceeds and to predict the outcome. Kinetic modeling translates a chemical reaction mechanism into constituent differential equations through the application of the law of mass action [21,22]. 

For the periodate oxidation of cellulose, kinetic modeling could provide reaction-important input for informed process design to maximize the yield of DAC. However, to date, there is little information available regarding rate constants concerning the kinetics of periodate oxidation of cellulose. Our aim was, therefore, to revisit and elaborate on the reaction kinetics of periodate-mediated oxidation of cellulose, advancing the hypothesis that kinetic modeling helps to understand the rate-limiting steps and overall kinetics of the cellulose oxidation reaction, and hopefully provide valuable information for optimizing the process toward a faster reaction. Due to the complexity of the reaction process and difficulties in obtaining quantitative values of the degree of oxidation, a lumped-parameter kinetic model of cellulose oxidation was used to predict oxidation reaction products and the rate constants. Kinetic modeling of periodate-mediated cellulose oxidation may also provide important insights into the process conditions that impact the efficiency of the reaction as well as the quality of the final products.

## 2. Materials and Methods

### 2.1. Materials

Never-dried, bleached, unbeaten, softwood kraft fibers were supplied by BillerudKorsnäs, Solna, Sweden, as the cellulose source. According to the supplier, the cellulose had a content of 80% glucose, 18.5% other carbohydrates, and 1.5% lignin as determined by carbohydrate analysis by the supplier. The chemicals needed during the oxidation steps, sodium (meta)periodate, isopropanol (≥99.8% purity), sodium hydroxide (NaOH), and the aldehyde content analyses reagent (hydroxylamine hydrochloride, NH_2_OH·HCl) were obtained from Sigma-Aldrich, Stockholm, Sweden. All the reagents were of analytical grade and used without further purification.

### 2.2. Synthesis of DAC

To oxidize the cellulose to 2,3-DAC, 5.4 g of sodium periodate was added per gram of dry weight of fiber to a gently stirred 4 g/L fiber water suspension [23]. Isopropanol (6.3 vol% in water) was added to the suspension as a radical scavenger to prevent side reactions [24,25]. The fibers were allowed to react at room temperature and in the dark [26] for 12 h. The oxidation reaction was stopped by adding ethanol to the reaction vessel to quench the residual periodate. The residue was isolated by filtration and thoroughly washed with deionized water until a conductivity of <5 μS/cm was reached. The cellulose was resuspended in Milli-Q water and filtered with a nylon cloth. The experiment was replicated three times. 

### 2.3. Determination of Aldehyde Content

The quantity of aldehydes formed, i.e., the degree of oxidation, was determined by adding hydroxylamine hydrochloride [27]. The added hydroxylamine hydrochloride is assumed to react quantitatively with all available carbonyls. For each analysis, 25 mL of a 0.25 M solution of hydroxylamine hydrochloride (adjusted to pH 4) was mixed with about 0.1 g of fibers and stirred for 2 h. After 2 h, the fibers were filtered off and dried in an oven at 105 °C overnight for determination of dry weight. The filtrate was titrated back to pH 4 using 0.1 M sodium hydroxide [28]. The degree of oxidation was calculated from the amount of sodium hydroxide required for the titration. Three separate reactions with hydroxylamine were carried out for each sample.

### 2.4. Fourier-Transform Infrared Spectroscopy, FTIR

The Fourier-transform infrared (FTIR) spectra were obtained on a PerkinElmer Spectrum One Spectrometer (PerkinElmer Instruments, Waltham, MA, USA). Triplicates of cellulose samples of approximately 2 mg were prepared by mixing with 200 mg of spectroscopic grade KBr. Spectra were recorded from 400 to 4000 cm^−1^ with a resolution of 4 cm^−1^. Spectra were calculated means of 16 individual scans per sample.

### 2.5. Field Emission Scanning Electron Microscopy, FE-SEM

Morphological analysis of cellulose and DAC was performed by FE-SEM analysis using a Hitachi S-4800 (Spectral Solutions, Solna, Sweden), equipped with an EDX (energy dispersive X-ray) detector for chemical analysis, and an EBSD (electron backscattered diffraction) detector for grain orientation and texture analysis. The samples were coated with a thin layer of gold (4 nm) using a Cressington sputter coater 208HR (Cressington Scientific Instruments Ltd., Watford, U.K.) and analysis was performed with SE In-Lens, under a high vacuum and with an operating voltage of 0.1–30 kV and a probe current of 10 nA.

### 2.6. Contact Angle Measurements

Films of DAC were prepared by filtering suspended DAC in water solution using a cutout of nylon cloth over a funnel and drying them overnight. There was no pressing or ironing of the sheets during the procedure. The contact angle (θ_C_) of cellulose and DAC films was measured using a setup composed of a charged coupled device (CCD) camera and adjustable background lighting under controlled temperature (23 °C) and relative humidity (50%). The measurements were performed with 8 μL drops of distilled water, applied by a manual precision dosage. The angle between the water drop and the surface was determined with One Attension software (Attension Optical Tensiometer, Biolin Scientific, Västra Frölunda, Sweden). The θ_C_ measurement was started 10 s after placing the droplet on the surface of each film, respectively. The measurements were performed in triplicates.

### 2.7. Design of Oxidation Experiments for Kinetic Modeling

The effects of individual variables (IO_4_^−^ and temperature) on the formation of DAC and the simultaneous degradation of cellulose and DAC were determined by varying one variable at a time. The total degradation was quantified as the loss of fibers (L), calculated from the difference in content of cellulose fibers after (w_a_) and before (w_b_) the reaction, where L = w_b_ − w_a_.

The details of the design of experiments (DOE) and the variations within oxidizing agent (IO_4_^−^) concentration and temperature are given in Table 1. All reactions were performed in triplicates.

## 3. Results and Discussion

### 3.1. Characterization of the Synthesized DAC

**Figure 1:**
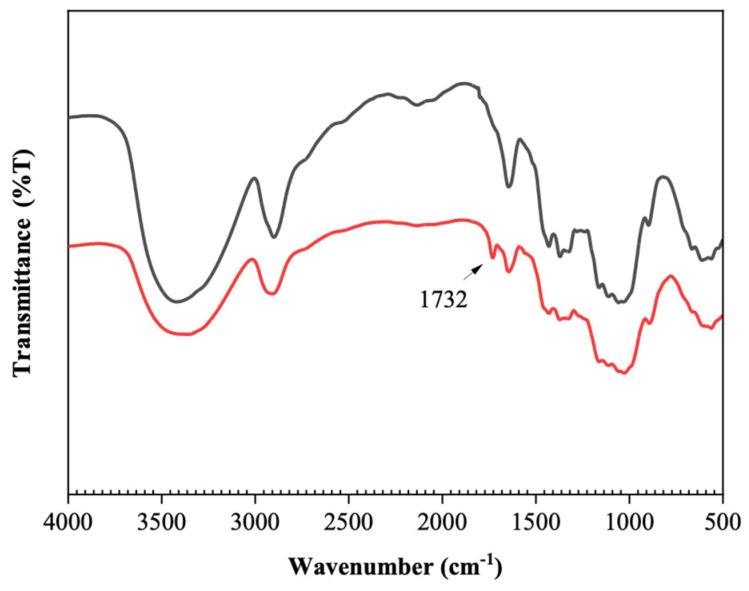
FTIR spectra of cellulose (black) and DAC (red) with a degree of oxidation of 40%, prepared at 25 °C and with an IO_4_^−^ concentration of 1 mol/L.

Structural characterization with FTIR reveals the changes in functional groups of cellulose after oxidation (Figure 1). The main bands in all spectra can be assigned as follows [19,29,30]: 3100–3500 cm^−1^ (OH stretching, H-bond), 2900–2945 cm^−1^ (C-H stretching of CH_2_ and CH groups), 1734 cm^−1^ (C=O stretching vibration), 1654 cm^−1^ (water OH bending vibration), 1429 cm^−1^ (CH_2_ symmetric bending vibration), 1373 cm^−1^ (C-H bending vibration), 1167 cm^−1^ (anti-symmetric bridge C-O-C stretching vibration), 1024 cm^−1^ (C-O-C stretching vibration, ring deformation vibration), and 890–860 cm^−1^ (CH deformation vibration of unite rings and (C=O) H). A hemiacetal band is observed at 875 cm^−1^. After a 2 h reaction, a noticeable peak at 1732 cm^−1^ arises, which indicates the formation of C=O, signifying the oxidation of cellulose to dialdehyde cellulose. Simultaneously, the broad O-H stretching band weakened in intensity.

The degree of oxidation was calculated from titration and shown to vary with both reaction temperature and the concentration of IO_4_^−^. The degree of oxidation obtained at 25 °C (0.1–1.0 mol/L) increased from 25.5% to 33% with increasing periodate concentration at the longest reaction time (10 h). At a higher temperature (55 °C), the maximum degree of oxidation obtained was 44.5% at a periodate concentration of 0.5 mol/L. The effect of temperature is more pronounced than the effect of periodate concentration. The degree of oxidation of DAC obtained after 4 h at 55 °C was 26%, which was fast oxidation compared to the reaction at 25 °C, reaching only a degree of oxidation of 30% after 10 h. 

### 3.2. Morphology of DAC and Cellulose Fibers

**Figure 2:**
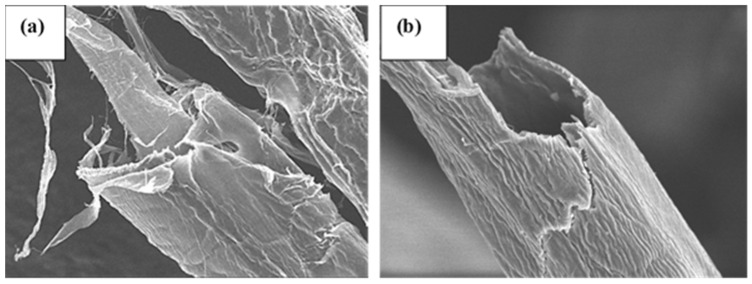
Scanning electron micrographs of fibers at a magnification of 2500: (**a**) pulp; (**b**) DAC with a degree of oxidation of 40%.

The DAC fibers, when analyzed using SEM, exhibited a smooth surface (Figure 2b), while pristine pulp fibers displayed a rough, woody surface (Figure 2a). SEM micrographs indicate the shrinkage and compaction of the fiber surface upon oxidation and the surface appeared to be free from pores on the micrometer scale. Oxidized samples seem to form smoother and more compact structures than the corresponding non-modified fibers as indicated by the films prepared from DAC. The results align with the findings of Plappert et al. who documented the absence of macroscopic and sub-microscopic pores within DAC films [31]. Such uniformity and smoothness may be the result of the disintegration of crystalline parts of cellulose caused by periodate oxidation [2]. Such disintegration has been demonstrated to support the formation of intra- and intermolecular cross-linking via hemiacetal and hemialdal linkages which would support the mechanical integrity of the material [32].

**Figure 3:**
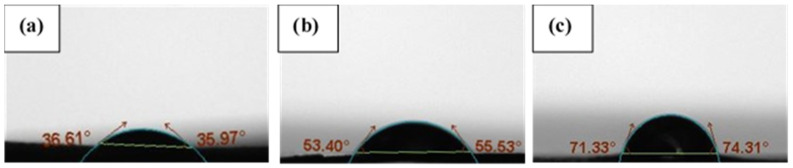
The contact angle of films made from DAC fibers prepared by oxidation for (**a**) 8 h, degree of oxidation = 24.5%; (**b**) 12 h, degree of oxidation = 32.5%; (**c**) 16 h, degree of oxidation = 40%.

The contact angle (θ_C_) of water on pulp and DAC film surfaces was measured 10 s after the droplet had been in contact with the surface. θ_C_ = 0 for the pristine pulp films and increased with a longer reaction time. The contact angle increased from 36° (8 h of oxidation), to 53° (12 h), and 71° (16 h) (Figure 3). The increase in θ_C_ indicates that high oxidation times cause the hydrophobicity to increase, as expected. A reduction in the amount of hydroxyl groups and an increase of more hydrophobic aldehyde groups give the films a higher hydrophobicity.

### 3.3. Cellulose Oxidation Kinetics

**Figure 4:**
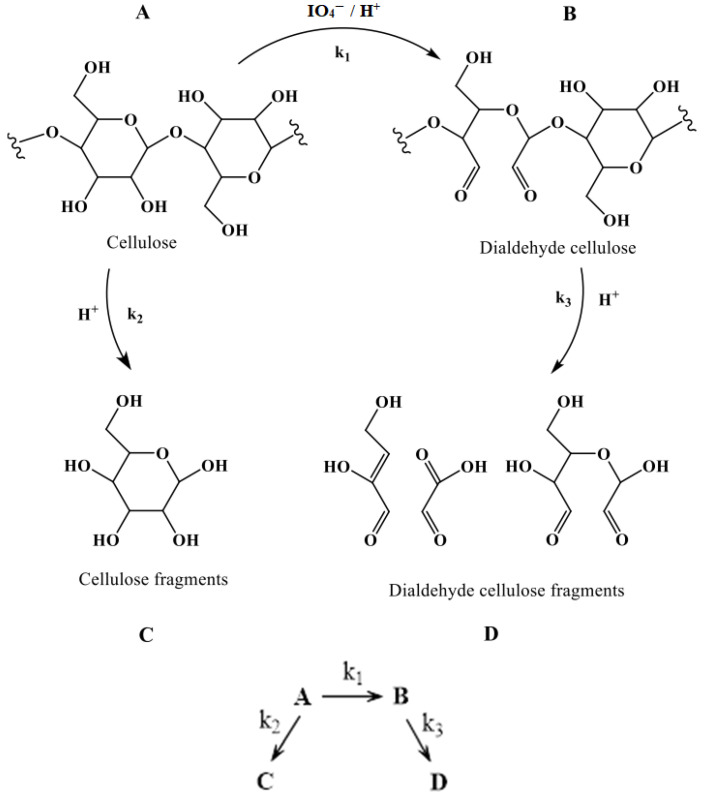
A schematic of periodate-mediated oxidation of cellulose. Parallel degradation reactions and associated rate constants occurring during cellulose oxidation. (**A**) Represents glucose units of a cellulose chain in the fiber, (**B**) represents 2,3-dialdehyde units of an oxidized cellulose chain, and (**C**,**D**) represent oligoglucans obtained by the degraded cellulose and dialdehyde cellulose, respectively.

DAC is obtained by oxidative cleavage of carbon–carbon bonds in the anhydro-D-glucopyranose residues under acidic conditions. Specifically, periodate mediates a cleavage of the C2–C3 bond, forming a periodate ester which rearranges into an aldehyde group [2]. The generated aldehyde functionalities are quite reactive and can take the form of fully hydrated aldehydes. Inter- and intramolecular hemiacetal formation between C2/C6 and C3/C6 in the glucoside units is also possible [10]. Concomitant degradation of cellulose as the degree of oxidation increases has been reported, leading to non-uniform oxidation products, the formation of shorter cellulose fragments, and ultimately the successive loss of cellulose [30,33,34,35,36]. The acidic reaction medium causes degradation of the both cellulose and oxidized segments by hydrolytic cleavage of β-1-4-glycosidic bonds [35,36] and the dialdehyde form is considered more susceptible to hydrolytic degradation compared to unmodified cellulose. Collectively, degradation was postulated to proceed both via beta-cleavage and acid hydrolysis of the glycosidic bonds between the cellulose units. Hence, at least two simultaneous degradation reactions must be considered and the total system—periodate-mediated oxidation of cellulose and concurrent degradation depicted in Figure 4—can, therefore, be translated into a series of parallel reactions.

The reactions are assumed to occur on the surface of fibers and fibrils so we consider it as a solid–liquid heterogeneous reaction. The activity—effective concentration—of cellulose or dialdehyde cellulose depends on the accessible parts on the surface of cellulose and the acidity. Thus, it is assumed that the oxidation reaction is pseudo-first order. The degradation reaction is pseudo-zero order. Periodate is always present in the solution in large excess, and its activity can therefore be considered to not change throughout the experiment. A lumped-parameter pseudo-homogeneous oxidation kinetics approach was used for the calculations as the reaction can be viewed as a system in which the spatial variations are not significant. Simplicity and easy implementation motivate the choice of such an approach compared to, for instance, the distributed-parameter model which incorporates concentration or temperature gradients along the reactor [21].

**Figure 5:**
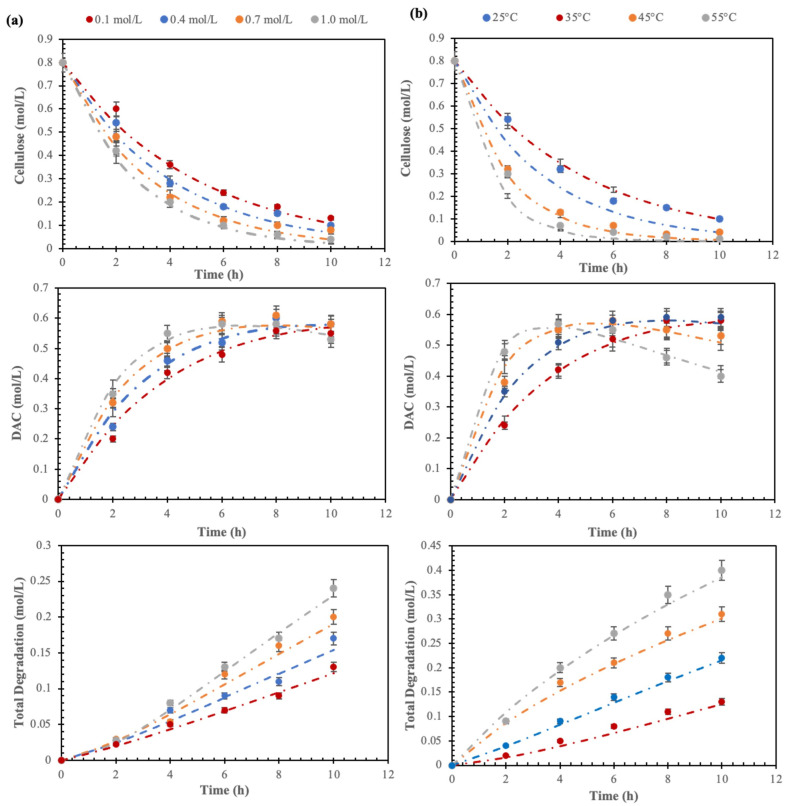
Time-variant experimental and model-predicted molar concentrations of cellulose, DAC, and total degradation (loss of fibers) under varying: (**a**) IO_4_^−^ concentration (0.1–1.0 mol/L) at 25 °C; (**b**) temperature (25–55 °C) at 0.5 mol/L. Each dotted line represents the trends predicted by the model and each symbol represents an experimental data point.

The overall reaction is first divided to individual sub-reactions and each step is quantified individually, providing a set of rate equations. Therefore, the rate equations relating to the molar concentration of A, B, and degraded species (Deg) (Figure 5) are given by the ordinary differential Equations (1a)–(1c) related to the direct rate law of a chemical reaction involving the reagents A and B:

*d* C_A_/*d*t = − k_1_C_A_ − k_2_C_A_ (1a)

*d* C_B_/*d*t = k_1_C_A_ − k_3_C_B_ (1b)

*d* C_Deg_/*d*t = k_2_C_A_ + k_3_C_B_ (1c)

where C_A_, C_B_, and C_Deg_ are the molar concentrations of A, B, and degraded products (C + D), respectively. k_1_ is the rate constant for the formation of dialdehyde cellulose, and k_2_ and k_3_ are the degradation constants of cellulose, A in Figure 4, and dialdehyde cellulose, B in Figure 4, respectively.

When solving Equations (1a)–(1c) using the initial conditions (i.e., C_A_ = C_A,0_, C_B_ = 0, C_C_ = 0, and C_Deg_ = 0 at t = 0), the following time-variant concentration profiles of A, B, and (C + D) were obtained by differentiating Equations (1a)–(1c):

(2a) $$C_{A}=C_{A,0}e^{-\left(k_{1}+k_{2}\right)t}$$

(2b) $$C_{B}=\frac{k_{1}C_{A,0}}{k_{3}-\left(k_{1}+k_{2}\right)}\left(e^{-\left(k_{1}+k_{2}\right)t}-e^{-k_{3}t}\right)$$

(2c) $$C_{Deg}=\frac{k_{1}k_{3}C_{A,0}}{k_{3}-\left(k_{1}+k_{2}\right)}\left\{\frac{e^{-k_{3}t}}{k_{3}}-\frac{e^{-\left(k_{1}+k_{2}\right)t}}{\left(k_{1}+k_{2}\right)}\right\}+\frac{k_{1}k_{3}C_{A,0}}{\left(k_{1}+k_{2}\right)k_{3}}+\frac{k_{2}C_{A,0}}{\left(k_{1}+k_{2}\right)}\left(1-e^{-\left(k_{1}+k_{2}\right)t}\right)$$

(2d) $$C_{B}=f\left(C_{A}\right)$$

Upon differentiation of Equation (2b) and setting it to zero, a maximum concentration of B and corresponding time were obtained and given by Equations (3) and (4):

(3) $$C_{B,max}=\frac{k_{1}C_{A,0}}{\left(k_{1}+k_{2}\right)}{\left(\frac{k_{1}+k_{2}}{k_{3}}\right)}^{\frac{k_{3}}{k_{3}-\left(k_{1}+k_{2}\right)}}$$

(4) $$t_{B,max}=\frac{1}{k_{3}-\left(k_{1}+k_{2}\right)}ln\left(\frac{k_{3}}{\left(k_{1}+k_{2}\right)}\right)$$

Therefore, knowing C_B,max_ and t_B,max_ from all time-variant isothermal B concentration profiles (Figure 5), k_1_, k_2_, and k_3_ were determined for all DOEs (variation in IO_4_^−^ concentration and temperature). The details of the observed k_1_, k_2_, and k_3_ are given in Table 2, respectively. The intrinsic rate constants [i.e., k_10_(T) k_20_(T), and k_30_(T)] at the given temperature were evaluated separately by knowing one pair of k_1_, k_2_, and k_3_ data using any DOE.

### 3.4. Synthesis Parameter Impact on Cellulose Oxidation

Glycosidic bonds between units of cellulose are cleaved under acidic conditions, which results in the degradation of cellulose with subsequent fiber loss. However, it was reported that a pH > 4 would inhibit the periodate oxidation of cellulose [20]. Therefore, we considered pH 3–4 to be a suitable acidity in our experiments for preparing 2,3-dialdehyde cellulose. 

The impact of IO_4_^−^ concentration and temperature on the oxidation of cellulose to DAC and its subsequent degradation is illustrated in the graphs in Figure 5, where the dotted lines represent the trends predicted by the model and each symbol represents an experimental data point. These graphs display the variations in cellulose and DAC molar concentrations at different IO_4_^−^ concentrations and temperatures. The IO_4_^−^ concentration and temperature both have a marked effect on DAC formation and total degradation. It was observed that the maximum yield of DAC was achieved in less time by increasing the periodate concentration, whereas the total degradation increases linearly with time. This is probably due to the structural change of the cellulose fibers, being partially degraded by the periodate at higher concentrations. The periodate-mediated oxidation of cellulose is considered to be a self-accelerating process [2] and the concomitant decrease in the degree of crystallinity improved the accessibility and reactivity of cellulose fiber surfaces. Thus, while the concentration of periodate increases, the aldehyde content increases and influences the process of cellulose degradation at the initial stage but reaches a maximum after some time. On the other hand, the rate constants (k_1_, k_2_, and k_3_ in Figure 4 and Table 2) increased considerably at elevated temperatures resulting in increased formation of DAC and with simultaneous degradation and fiber loss, quantified by yield. This could be explained by the Arrhenius equation (k = A e^(−Ea/RT)^), which gives the relation between k and T. Temperature increase of 10 °C resulted in an approximately 5% increase in DAC formation and a 15% increase in degradation.

Figure 5 depicts the molar concentration profiles, both the experimental data and the relationships predicted by the model, of cellulose, DAC, and total degradation under varying (a) IO_4_^−^ concentration (0.1–1.0 mol/L) at 25 °C, and (b) temperature (25–55 °C) at 0.5 mol/L. The experimental concentration profiles were used to determine the rate constants (k_1_, k_2_, and k_3_) and confirm the pseudo-first-order oxidation reaction, using integral Equations (2a)–(2d). The details of k_1_, k_2_, and k_3_ are presented in Table 2. It is interesting to see that k_1_ is much higher than k_2_ and k_3_ (which are the rate constants of degradation of cellulose and dialdehyde cellulose, respectively), which signifies the conversion of dialdehyde cellulose as the rate-determining step. It is observed that the increase of k_1_ with IO_4_^−^ concentration is less significant than the increase with temperature. It is furthermore noteworthy that k_2_ and k_3_ remain almost unaffected by the increase in IO_4_^−^ concentration but increase significantly with an increase in temperature. The concentration of periodate and temperature both have a marked effect on DAC formation and total degradation. The formation of DAC increased with increasing temperature but it comes at the cost of an even more prominent simultaneous degradation and fiber loss. Taken together, these findings show how to balance temperature and periodate concentration to maximize the output of DAC. The oxidation reaction is assumed to be pseudo-first order while the degradation reaction is pseudo-zero order. C_B,max_ and t_B,max_ were determined from all time-variant isothermal B concentration profiles, and k_1_, k_2_, and k_3_ were determined for all DOEs in Table 2.

**Figure 6:**
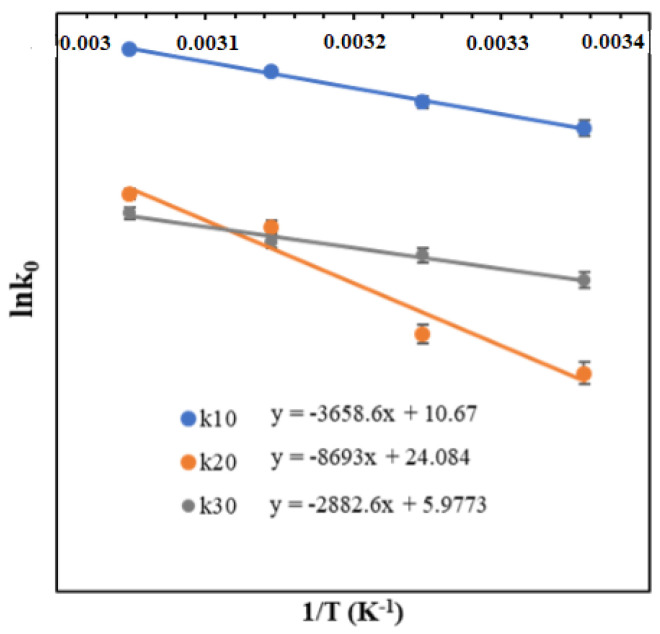
The variation of rate constants with temperature for the periodate-mediated oxidation of cellulose is calculated with the Arrhenius equation.

Temperature-dependent k_10_(T), k_20_(T), and k_30_(T) were determined along with the observed k_1_, k_2_, and k_3_ values, which were obtained from temperature-dependent DOEs. The calculated k_10_(T), k_20_(T) and k_30_(T) were fitted with the Arrhenius equation. The details of lnk_10_(T) versus 1/T and lnk_20_(T) and lnk_30_(T) versus 1/T are shown in Figure 6 and the corresponding Arrhenius equations for k_10_(T), k_20_(T), and k_30_(T) are given by Equations (5a)–(5c):

(5a) $$k_{10}\left(T\right)=4.36\times 10^{4}e^{-\frac{3858.6}{T}}$$

(5b) $$k_{20}\left(T\right)=2.8\times 10^{10}e^{-\frac{8693}{T}}$$

(5c) $$k_{30}\left(T\right)=3.94\times 10^{2}e^{-\frac{2882.6}{T}}$$

The presented findings on oxidation rate constants and temperature-dependent intrinsic kinetic constants bear significance in understanding the effects of reaction parameters on the successful synthesis of DAC. This could be useful in scaling and aid in process design, supporting the growing interest in oxidized cellulose derivatives as bio-based materials and as a viable platform for post-functionalization [13,37]. Current and emerging trends in this regard involve exploring DAC derivatives for biomedical applications [13] and water purification matrices [38,39]. The potential of generating diverse cellulosic nanoparticles via periodate-mediated oxidation is also attracting interest [40]. The ongoing industrial transition to greener chemistry is a strong driver to revisit the oxidation chemistry and process design. Reduction of process temperature, time, or periodate concentration would all be helpful in this regard. Periodate oxidation of nanocellulose under microwave irradiation was shown to proceed efficiently at room temperature [41]. Reduction of IO_4_^−^ and the regeneration of residual periodate from the DAC reaction mixture are also in the spotlight. Kinetic modeling of chemical reactions benefits largely from the rapid advances in automated computation and systems of high complexity are becoming assessable [22]. 

## 4. Conclusions

The oxidation of cellulose was investigated using various IO_4_^−^ concentrations and at different temperatures. Lumped-parameter pseudo-homogeneous oxidation kinetics of cellulose was modeled and temperature-dependent intrinsic kinetic constants, i.e., frequency factor and activation energy, were also determined. The experimental concentration profiles were used to determine the rate constants for oxidation of cellulose to DAC (k_1_) and the concomitant degradation of cellulose (k_2_) and DAC (k_3_), showing that k_1_ is consistently higher than k_2_ and k_3_ in the studied process window. The DAC formation followed pseudo-first-order reaction kinetics and displayed a significant rate increase at elevated temperatures, resulting in a faster formation of DAC but also a faster simultaneous degradation. A temperature increase of 10 °C resulted in an approximately 5% increase in DAC formation and a 15% increase in degradation. However, the rate of DAC formation (k_1_) also increased with IO_4_^−^ concentration, while k_2_ and k_3_ remained largely unaffected. Therefore, parameter-dependent variation of rate constants quantified in this work provides important input for informed process design to maximize the yield of DAC. 

The energy of activation for k_10_, k_20_, and k_30_ were determined and found to be 30.4 kJ mol^−1^, 72.27 kJ mol^−1^, and 23.9 kJ mol^−1^, respectively, indicating the extent of sensitivity of cellulose oxidation and dialdehyde cellulose degradation with temperature. The proposed kinetic model under varying temperatures and IO_4_^−^ concentration fitted well with the time-variant experimental concentration profile, which can be used for the scale-up of the process. Overall, kinetic modelling of cellulose oxidation provides a valuable tool for advancing our understanding of the reaction and for optimizing the production of cellulose-based materials and chemicals.

## Figures and Tables

**Table 1:** Experimental design.

Experiment Number	Temperature (°C)	Concentration of IO_4_^−^ (mol/L)
1		0.01
2	25	0.04
3		0.07
4		1.0
5	25	
6	35	0.5
7	45	
8	55	

**Table 2:** Rate constants under varying reaction conditions.

	Temperature (°C)	Concentration (mol/L)	k_1_ (h^−1^)	k_2_ (h^−1^)	k_3_ (h^−1^)
IO_4_^−^ concentration variation		0.01	0.1896	0.0110	0.0213
		0.04	0.2357	0.0128	0.0270
	25	0.07	0.2893	0.0135	0.0352
		1.0	0.3475	0.0165	0.0420
Temperature variation	25		0.2363	0.0108	0.0281
	35	0.5	0.2910	0.0117	0.0353
	45		0.4441	0.0519	0.0421
	55		0.6121	0.0809	0.0630

## Data Availability

Data are contained within the article.
